# Associations of dietary fiber knowledge and intake with glycemic control among Jordanian patients with type two diabetes mellitus: a cross-sectional study

**DOI:** 10.29219/fnr.v70.13819

**Published:** 2026-08-31

**Authors:** Dana Abu Hamed, Refat Ahmed Alkurd, Mousa Abujbara, Yousef Khader, Kamel M. Ajlouni

**Affiliations:** 1 The National Center for Diabetes, Endocrinology, and Genetics (NCDEG), Amman, Jordan; 2 Department of Nutrition, Faculty of Pharmacy and Medical Sciences, University of Petra, Amman, Jordan; 3 Department of Public Health, Jordan University of Science and Technology (JUST), Irbid, Jordan

**Keywords:** dietary fiber, HbA1c, type 2 diabetes mellitus, nutrition knowledge, Jordan

## Abstract

**Background:**

Dietary fiber plays an important role in glycemic control, as reflected by glycated hemoglobin (HbA1c), among individuals with type 2 diabetes mellitus. However, evidence from Jordan regarding patients’ knowledge of dietary fiber, consumption patterns, and perceived barriers to adequate intake remains limited.

**Objective:**

This study aimed to assess dietary fiber knowledge and consumption frequency among patients with type 2 diabetes mellitus, and to examine their associations with glycemic control and perceived barriers to adequate intake.

**Methods:**

A cross-sectional study was conducted among 722 adults with type 2 diabetes mellitus attending the National Center for Diabetes, Endocrinology, and Genetics in Amman, Jordan. Data were collected using a validated questionnaire that assessed dietary fiber knowledge related to food sources and health benefits (15 items), frequency of consumption of fiber-rich foods, and perceived barriers to intake. Glycemic control was evaluated using HbA1c levels.

**Results:**

Most fiber-rich food items showed no statistically significant association with glycated hemoglobin levels (*P* > 0.05); however, oat consumption was significantly associated with better glycemic control. Higher dietary fiber knowledge was significantly associated with improved glycemic control, both in relation to knowledge of health benefits (*P* = 0.049) and food sources (*P* = 0.017). Despite this, 61.9% of participants had suboptimal glycemic control, with HbA1c levels of 7–8% or > 8%. Gastrointestinal discomfort and lack of awareness were the most commonly reported barriers to adequate dietary fiber intake.

**Conclusion:**

Better glycemic control among patients with type 2 diabetes mellitus is associated with higher dietary fiber knowledge; however, existing barriers may hinder the translation of knowledge into actual dietary practices. Educational interventions should emphasize practical and culturally appropriate strategies to support the incorporation of dietary fiber into daily diets. Future research should focus on closing the gap between dietary fiber knowledge and its effective application in diabetes management.

## Popular scientific summary

Dietary fiber may help people with type 2 diabetes control blood sugar. This study included 722 adults with type 2 diabetes in Jordan. Better dietary fiber knowledge was linked to better glycemic control, but many patients still had high HbA1c levels. Common barriers included digestive discomfort and lack of awareness. Practical nutrition education is needed to help patients include dietary fiber in daily meals.

Type 2 diabetes mellitus is a chronic metabolic disorder marked by elevated blood glucose levels and impaired insulin function, resulting from either insufficient insulin secretion or decreased effectiveness of insulin. Over time, uncontrolled diabetes can cause serious complications affecting the heart, blood vessels, eyes, kidneys, and nerves (1, 2). According to the 11th edition of the International Diabetes Federation (IDF) Diabetes Atlas: 589 million adults aged 20–79 years in 2024, projected to increase to 853 million by 2050 (3). In Jordan, the prevalence of Type 2 diabetes mellitus is notably high and continues to rise, with reported rates of 18.1% among women and 32.4% among men in 2017 (4). Embracing a healthy lifestyle and balanced dietary habits is essential for the prevention and management of chronic diseases, especially Type 2 diabetes mellitus (5).

A balanced diet and a healthy lifestyle are crucial for managing and preventing long-term diseases (5). In this context, dietary fiber plays an important role in glycemic regulation through its effects on glucose metabolism, making it a key dietary component in the management of type 2 diabetes mellitus (6). Dietary fiber is categorized as either soluble or insoluble, and each has unique physiological activities and physicochemical traits (7). Dietary fiber is naturally present in cereals, fruits, vegetables, and nuts (8). Consequently, sufficient consumption of dietary fiber has been associated with enhanced insulin sensitivity (9), diminished postprandial hyperglycemia (10), decreased cholesterol and triglyceride levels (11, 12), lowered blood pressure (13), reduced systemic inflammation (14), and assistance in weight management (15).

Current dietary guidelines recommend a daily intake of at least 14 g of dietary fiber per 1,000 kcal (7). However, evidence from the Jordanian population indicates actual fiber intake remains below recommended levels, with average consumption of only 24.4 g per day, equivalent to roughly 8 g per 1,000 kcal, which represents nearly two-thirds of the recommended intake (16). This insufficient intake may limit the potential metabolic benefits of dietary fiber, especially among individuals with type 2 diabetes mellitus.

Despite the well-established role of dietary fiber in glycemic regulation, existing evidence has largely examined individual aspects of fiber intake in isolation. Limited attention has been given to a more comprehensive understanding that considers dietary fiber knowledge, food sources, consumption patterns, and real-life barriers together, particularly among patients with type 2 diabetes mellitus. Moreover, studies adopting an integrated approach that reflects everyday dietary practices remain scarce. This gap highlights the need to explore how these interrelated factors collectively influence glycemic control in real-world settings. Therefore, this study aimed to assess the level of knowledge regarding dietary fiber among patients with type 2 diabetes mellitus, to evaluate the frequency of consumption of dietary fiber-rich foods, to examine the association of dietary fiber knowledge and consumption frequency with glycated hemoglobin levels, and to identify perceived barriers that limit dietary fiber intake in this population.

## Materials and methods

### Study design and settings

This cross-sectional study was conducted from December 2024 to February 2025 among patients with Type 2 diabetes mellitus attending the National Center for Diabetes, Endocrinology, and Genetics (NCDEG) in Amman. This design is suitable for estimating the knowledge and consumption of dietary fiber. The study protocol was reviewed and approved by the Institutional Review Board (IRB) of the National Center for Diabetes, Endocrinology and Genetics (NCDEG), Amman, Jordan (Approval No. 1/2025; Approval Date: January 7, 2025).

### Participants

The study included 722 patients with Type 2 diabetes mellitus of both genders who were 18 years of age or older and capable of giving informed consent.

### Eligibility criteria

The study population consisted of adult men and women aged 18 years or older who had been diagnosed with type 2 diabetes mellitus and were receiving follow-up care at the NCDEG. Eligible participants were those managed with insulin, oral hypoglycemic agents, or a combination of both, and who were able to provide informed consent. Exclusion criteria included individuals younger than 18 years; those diagnosed with type 1 diabetes mellitus or an unspecified type of diabetes; pregnant women, including those with gestational diabetes; and individuals unable to comprehend the questionnaire or provide informed consent.

### Sampling and sample size

A systematic random sampling technique was used to select patients. After being informed of the study’s goal, every fifth patient who attended the reception and met the inclusion criteria was invited to participate.

The required sample size was calculated using the single proportion formula (17):

$$n=\frac{Z^{2}P(1-P)}{E^{2}}$$

Assuming 50% of the population follows the dietary behavior of interest. With a 95% confidence level (*Z* = 1.96) and a 5% margin of error (*E* = 0.05), the minimum sample size required was 385 participants. However, it was increased to 722 to enable subgroup analyses and ensure more representative results.

### Variables, data sources, and measurements

Glycemic control was the primary outcome variable and was assessed using HbA1c values. HbA1c was classified into three categories: less than 7%, 7–8%, and greater than 8%.

The main exposure variables included dietary fiber knowledge and the frequency of consumption of dietary fiber-rich foods. Dietary fiber knowledge was categorized based on the proportion of correct responses, with scores below 60% classified as poor knowledge, scores between 60 and 80% as moderate knowledge, and scores above 80% as good knowledge. Consumption of dietary fiber-rich foods was assessed according to self-reported frequency and categorized as daily, several times per week, one to two times per week, less than once per week or never.

Other variables collected included age, sex, body mass index, educational level, marital status, monthly income, duration of type 2 diabetes mellitus, presence of comorbidities, current medications, irritable bowel syndrome, constipation, and cardiovascular disease.

Sociodemographic and clinical data were obtained through participant interviews and medical record review. Sociodemographic information included age, sex, place of residence, marital status, educational attainment, occupation, and monthly income. Anthropometric measurements, including weight and height, were retrieved from medical records and used to calculate body mass index. Biochemical data consisted of the three most recent HbA1c measurements extracted from medical records. Clinical characteristics, including duration of type 2 diabetes mellitus, family history of diabetes, disease-related complications, and current medications, were obtained from both participant self-reports and medical records.

Consumption of dietary fiber sources was assessed by asking participants to report the frequency of consumption of a list of fiber-rich foods. Response options included ‘do not consume’, ‘less than once per week’, ‘one to two times per week’, ‘three times per week’, and ‘daily’.

Dietary fiber knowledge was assessed through questions addressing knowledge of fiber-rich food sources, food preferences when eating outside the home, and awareness of the relationship between dietary fiber intake and health conditions, including cardiovascular disease, obesity, diabetes, and hypercholesterolemia.

Perceived barriers to dietary fiber intake were evaluated by asking participants whether they considered high-fiber foods to be expensive, of limited health benefit, unpleasant in taste, or difficult to access.

The questionnaire was pre-tested on a small group of patients (*n* = 20) with type 2 diabetes mellitus attending the study center to assess clarity, comprehension, and completion time. Minor wording modifications were made based on participants’ feedback. In addition, internal consistency of the dietary fiber knowledge items was assessed, yielding an acceptable Cronbach’s alpha value (α = 0.78), indicating good reliability.

### Statistical analysis

Data were analyzed using the Statistical Package for the Social Sciences (SPSS), version 21. Continuous variables are expressed as means with standard deviations (SD), and categorical variables as frequencies and percentages. Individuals with missing information were excluded before the final sample was formed; therefore, no missing data were present in the analyzed dataset. The chi-square (*χ*^2^) test was used to assess associations between dietary factors (dietary fiber) and other variables. In addition, multivariable analyses did not reveal any statistically significant associations; therefore, the results are presented as bivariate analyses. A *P*-value of < 0.05 was considered statistically significant.

## Results

### Participants’ characteristics

Table 1 shows a total of 722 participants that were enrolled in the study, including 387 females (53.6%) and 335 males (46.4%), with a mean age of 62.7 ± 9.6 years. Most participants were married (81.3%) and lived in urban areas (82.8%). Regarding education, 40.7% had university or postgraduate degrees, while nearly half were retired or unemployed. The mean body mass index (BMI) was 31.3 ± 5.8 kg/m^2^, with obesity (BMI ≥ 30 kg/m^2^) observed in 54.7% of the sample, more prevalent among females (63.3%) than males (44.8%) (*P* < 0.001).

**Table 1:** Sociodemographic characteristics of diabetic patients (*N* = 722)

Variables	Total n* (%) 722	Male *n* (%) 335	Females *n* (%) 387	*P*
**Age, years, mean ± SD**	62.68 ± 9.590	62.31 ± 10.504	63.00 ± 8.723	
< 60	258 (35.7%)	125 (37.3%)	133 (34.4%)	0.413
60–65	180 (24.9%)	76 (22.7%)	104 (26.9%)	
> 65	284 (39.3%)	134 (40.0%)	150 (38.8%)	
**Body mass index (kg/m^2^), mean ± SD**	31.26 ± 5.79	29.96 ± 5.1	32.38 ± 6.11	
Normal < 25	99 (13.7%)	56 (16.7%)	43 (11.1%)	< 0.001
Overweight 25–30	228 (31.6%)	129 (38.5%)	99 (25.6%)	
Obese 30+	395 (54.7%)	150 (44.8%)	245 (63.3%)	
**Living**				
Urban	598 (82.8%)	281 (83.9%)	317 (81.9%)	0.484
Rural	124 (17.2%)	54 (16.1%)	70 (18.1%)	
**Marital status**				
Single	20 (2.8%)	5 (1.5%)	15 (3.9%)	< 0.001
Married	587 (81.3%)	318 (94.9%)	269 (69.5%)	
Divorced + widow	115 (15.9%)	12 (3.6%)	103 (26.6%)	
**Educational level**				
Less than high school	133 (18.4%)	29 (8.7%)	104 (26.9%)	< 0.001
High school	150 (20.8%)	62 (18.5%)	88 (22.7%)	
Diploma	145 (20.1%)	53 (15.8%)	92 (23.8%)	
University	214 (29.6%)	130 (38.8%)	84 (21.7%)	
Master or PHD	80 (11.1%)	61 (18.2%)	19 (4.9%)	
**Occupation**				
Employee	134 (18.6%)	104 (31.0%)	30 (7.8%)	< 0.001
Retired	351 (48.6%)	218 (65.1%)	133 (34.4%)	
House-wife/unemployed	237 (32.8%)	13 (3.9%)	224 (57.9%)	
**Income, JD**				
< 250	120 (16.6%)	27 (8.1%)	93 (24.0%)	< 0.001
250–500	294 (40.7%)	113 (33.7%)	181 (46.8%)	
500–800	180 (24.9%)	99 (29.6%)	81 (20.9%)	
> 800	128 (17.7%)	96 (28.7%)	32 (8.3%)	

*n* = number.

### Clinical characteristics of diabetic patients

Table 2 shows that 34.1% of participants had diabetes for less than 10 years, while 36.7% had the disease for ≥ 15 years. Glycemic control was suboptimal, with only 38.1% achieving HbA1c < 7%, while the remainder were classified as 7–8% or > 8%.

**Table 2:** Clinical characteristics of diabetic patients

Variables	Total *n* (%) 722	Males *n* (%) 335	Female *n* (%) 387	*P*
**Duration of diabetes**				
< 10 years	246 (34.1%)	109 (32.5%)	137 (35.4%)	0.606
10–15	211 (29.2%)	97 (29.0%)	114 (29.5%)	
≥ 15 years	265 (36.7%)	129 (38.5%)	136 (35.1%)	
**Family history of diabetes**				
Yes	556 (77.0%)	245 (73.1%)	311 (80.4%)	0.210
No	166 (23.0%)	90 (26.9%)	76 (19.6%)	
**HbA1c levels**				
< 7%	275 (38.1%)	129 (38.5%)	146 (37.7%)	0.831
7–8%	217 (30.1%)	103 (30.7%)	114 (29.5%)	
> 8%	230 (31.9%)	103 (30.7%)	127 (32.8%)	
**Hypertension**				
Yes	556 (77.0%)	254 (75.8%)	302 (78.0%)	0.481
No	166 (23.0%)	81 (24.2%)	85 (22.0%)	
**Cardiovascular disease**				
Yes	252 (34.9%)	139 (41.5%)	113 (29.2%)	0.001
No	470 (65.1%)	196 (58.5%)	274 (70.8%)	
**Dyslipidemia**				
Yes	609 (84.3%)	274 (81.8%)	335 (86.6%)	0.078
No	113 (15.7%)	61 (18.2%)	52 (13.4%)	
**Constipation**				
Yes	139 (19.3%)	50 (14.4%)	89 (23.0%)	0.006
No	583 (80.7%)	285 (85.1%)	298 (77.0%)	
**Diarrhea**				
Yes	40 (5.5%)	14 (4.20%)	26 (6.7%)	0.137
No	682 (94.5%)	321 (95.8%)	361 (93.3%)	
**Irritable bowel syndrome**				
Yes	212 (29.4%)	74 (22.1%)	138 (35.7%)	0.000
No	510 (70.6%)	261 (77.9%)	249 (64.3%)	
**Osteoporosis**				
Yes	127 (17.6%)	12 (3.6%)	115 (29.7%)	0.000
No	595 (82.4%)	323 (96.4%)	272 (70.3%)	
**Diabetes Medication**				
Oral Antidiabetic Agents	410 (56.8%)	190 (56.7%)	220 (56.8%)	0.937
Insulin	24 (3.3%)	12 (3.6%)	12 (3.1%)	
Oral Antidiabetic Agents + Insulin	288 (39.9%)	133 (39.7%)	155 (40.1%)	

Most patients were treated with oral antidiabetic medications (56.8%), while 39.9% used a combination of oral agents and insulin. Hypertension and dyslipidemia were highly prevalent (77.0 and 84.3%, respectively). Gender differences were observed: men had more cardiovascular disease (41.5% vs. 29.2%, *P* = 0.001), whereas women showed higher prevalence of osteoporosis (29.7% vs. 3.6%, *P* < 0.001), irritable bowel syndrome (35.7% vs. 22.1%, *P* < 0.001), and constipation (23.0% vs. 14.4%, *P* = 0.006).

### Consumption frequency of dietary fiber-rich foods and their association with HbA1c

Table 3 present the frequency of consumption of various dietary fiber-rich foods and their association with HbA1c. In general, most questioned food items, including cooked vegetables, salads, fruits with peel, whole grain bread, white bread, nuts, dried fruits, fruit juice, and cereals, showed no statistically significant association with HbA1c levels (*P* > 0.05). However, a notable exception was observed for oat consumption, which was assessed based on consumption frequency rather than portion size. Participants who reported consuming oats less than once per week or several times per week were significantly more likely to have controlled HbA1c levels (< 7%) compared with those in the higher HbA1c categories (7–8% and > 8%) (*P* = 0.005).

**Table 3:** Consumption frequency of dietary fiber-rich foods and their association with HbA1c

Food item	Frequency category	HbA1c < 7% *n* (%) 275	HbA1c ≥ 7% *n* (%) 447	Total *n* (%) 722	*P*
**Cooked vegetable**	Less than once a week	96 (34.4%)	183 (65.6%)	279 (38.6%)	0.057
	Several times a week	155 (39.4%)	238 (60.6%)	393 (54.4%)	
	Daily	21 (55.3%)	17 (44.7%)	38 (5.3%)	
	Never	3 (25.0%)	9 (75.0%)	12 (1.7%)	
**Salad -vegetables**	Less than once a week	27 (35.5%)	49 (64.5%)	76 (10.5%)	0.719
	Several times a week	117 (40.6%)	171 (59.4%)	288 (39.9%)	
	Daily	126 (36.6%)	218 (63.4%)	344 (47.6%)	
	Never	5 (35.7%)	9 (64.3%)	14 (1.9%)	
**Fruit peel**	Less than once a week	14 (29.8%)	33 (70.2%)	47 (6.5%)	0.126
	Several times a week	63 (32.8%)	129 (67.2%)	192 (26.6%)	
	Daily	193 (40.8%)	280 (59.2%)	473 (65.5%)	
	Never	5 (50.0%)	5 (50.0%)	10 (1.4%)	
**Whole grain bread**	Less than once a week	7 (30.4%)	16 (69.6%)	23 (3.2%)	0.553
	Several times a week	27 (34.6%)	51 (65.4%)	78 (10.8%)	
	Daily	105 (36.7%)	181 (63.3%)	286 (39.6%)	
	Never	136 (40.6%)	199 (59.4%)	335 (46.4%)	
**White bread**	Less than once a week	5 (19.2%)	21 (80.8%)	26 (3.6%)	0.134
	Several times a week	25 (32.5%)	52 (67.5%)	77 (10.7%)	
	Daily	127 (39.9%)	191 (60.1%)	318 (44.0%)	
	Never	118 (39.2%)	183 (60.8%)	301 (41.7%)	
**Nuts**	Less than once a week	90 (35.7%)	162 (64.3%)	252 (34.9%)	0.704
	Several times a week	102 (39.5%)	156 (60.5%)	258 (35.7%)	
	Daily	49 (41.2%)	70 (58.8%)	119 (16.5%)	
	Never	34 (36.6%)	59 (63.4%)	93 (12.9%)	
**Dried fruits**	Less than once a week	57 (36.1%)	101 (63.9%)	158 (21.9%)	0.615
	Several times a week	83 (41.7%)	116 (58.3%)	199 (27.6%)	
	Daily	98 (38.7%)	155 (61.3%)	253 (35.0%)	
	Never	37 (33.0%)	75 (67.0%)	112 (15.5%)	
**Fruit juice**	Less than once a week	75 (38.1%)	122 (61.9%)	197 (27.3%)	0.678
	Several times a week	53 (43.8%)	68 (56.2%)	121 (16.8%)	
	Daily	17 (34.0%)	33 (66.0%)	50 (6.9%)	
	Never	130 (36.7%)	224 (63.3%)	354 (49.0%)	
**Cereals**	Less than once a week	198 (38.4%)	318 (61.6%)	516 (71.5%)	0.591
	Several times a week	28 (38.4%)	45 (61.6%)	73 (10.1%)	
	Daily	4 (25.0%)	12 (75.0%)	16 (2.2%)	
	Never	45 (38.5%)	72 (61.5%)	117 (16.2%)	
**Oats**	Less than once a week	44 (50.6%)	43 (49.4%)	87 (12.0%)	0.005
	Several times a week	21 (55.3%)	17 (44.7%)	38 (5.3%)	
	Daily	8 (36.4%)	14 (63.6%)	22 (3.0%)	
	Never	202 (35.1%)	373 (64.9%)	575 (79.6%)	

### The reasons for the low consumption of dietary fiber

Table 4 demonstrates that barriers to dietary fiber intake were mainly related to side effects such as gases (23.4%), which were significantly more reported by females than males (*P* = 0.004). Moreover, limited availability (15.5%) was more commonly reported by females. Other barriers included insufficient awareness (23.5%), dislike of taste (19.5%), and high cost (10.2%), with no significant gender differences.

**Table 4:** The reasons for the low consumption of dietary fiber

Reasons for the lack of dietary fiber	All *n* (%)	Male *n* (%)	Female *n* (%)	*P*
**Reasons for the lack of dietary fiber**				
Side effects such as gases	169 (23.4%)	62 (18.5%)	107 (27.6%)	0.004
Availability	112 (15.5%)	42 (12.5%)	70 (18.1%)	0.040
I don’t like the dietary fiber taste	141 (19.5%)	63 (18.8%)	78 (20.2%)	0.648
Expensive	74 (10.2%)	31 (9.3%)	43 (11.1%)	0.412
Don’t know its importance	170 (23.5%)	82 (24.5%)	88 (22.7%)	0.583

### The association between the level of knowledge about dietary fiber food sources and glycemic control

Table 5 shows that both knowledge of fiber sources (*P* = 0.017) and health-related knowledge (*P* = 0.049) were significantly associated with HbA1c levels, where lower knowledge was linked to higher HbA1c.

**Table 5:** Association between the level of knowledge about dietary fiber and glycemic control (HbA1c) with dietary fiber health benefits and food sources, and glycemic control

Variable	HbA1c < 7% *n* (%)	HbA1c ≥ 7% *n* (%)	Total *n* (%)	*P*
**Dietary fiber health-related knowledge** *				
Poor knowledge	67 (32.4%)	140 (67.6%)	207 (28.7%)	0.049
Moderate knowledge	118 (41.0%)	170 (59.0%)	288 (39.9%)	
Good knowledge	90 (39.6%)	137 (60.4%)	227 (31.4%)	
**Knowledge of dietary fiber sources**				
Poor knowledge	116 (33.4%)	231 (66.6%)	347 (48.1%)	0.017
Moderate knowledge	129 (40.6%)	189 (59.4%)	318 (44.1%)	
Good knowledge	30 (52.6%)	27 (47.4%)	57 (7.9%)	

* Dietary fiber knowledge was classified as poor when fewer than 60% of questions were answered correctly, moderate when 60–80% were answered correctly, and good when more than 80% were answered correctly.

## Discussion

Dietary fiber has been recognized for improving glycemic control among patients with Type 2 diabetes mellitus; nevertheless, research on this specific topic in the Middle East, particularly Jordan, remains limited. This study evaluated dietary fiber intake (via frequency of dietary fiber-rich foods), HbA1c, and fiber-related knowledge in Jordanian patients, and it highlights a persistent gap between knowledge and everyday practice. Many studies showed a significant correlation between high fiber intake and lower HbA1c levels (18, 19).

In our study, 65.5% participants reported daily fruit intake, and no significant association was found between fruit intake and HbA1c. This result may be attributed to various factors, including common dietary practices in Jordanian culture of consuming several fruits a day, which may exceed recommendations (20, 21), and pairing them with added sugars or sweetened beverages (22), thereby reducing the glycemic benefits of fruit consumption. Changes in fruit intake may also coincide with changes in other dietary components, leading to a decrease in the metabolic effects of fruit (23). These findings are consistent with a meta-analysis showing fruit consumption reduced fasting glucose but not HbA1c (24), though other work reported higher odds of achieving HbA1c < 7% with fruit intake (25). Such inconsistency may reflect methodological differences, as our study used qualitative frequency rather than portion size, preparation methods, or co-consumption patterns.

Regarding bread, about (39.6% vs. 44.0%) reported consuming brown bread almost similar to proportion of white bread; proportions achieving HbA1c < 7% were comparable. A key contextual factor is that whole wheat bread in Jordan often consists of bleached flour with added bran, which lacks the most nutritious part of the grain, the wheat germ (26). This may explain the discrepancy with studies where genuinely whole grains reduced HbA1c (27, 28). Cultural food influences such as reliance on bread as the main carbohydrate source and frequent pairing of bread with high-fat foods may further diminish its benefits (29).

Vegetable intake, cooked or raw, also showed no significant association with HbA1c.

Trials reporting improvements typically specified effective thresholds ≥ 150 g/day total vegetables and ≥ 70 g/day green vegetables (30) or found inverse relations with raw vegetables (31). Differences in achieved portions, preparation styles or energy-dense accompaniments may account for the variations.

Oats were the only fiber-rich food significantly associated with glycemic control in this study. This finding may be partly attributed to the high content of soluble β-glucan in oats, which has been shown to slow glucose absorption and improve postprandial glycemic responses. Moreover, oat consumption may indicate healthier dietary behaviors, as individuals with better glycemic control may be more inclined to include oats in their diet; thus, reverse causation cannot be ruled out given the cross-sectional design (6, 32).

Overall, the lack of significant associations observed for most fiber-rich foods may be partly explained by the assessment of dietary intake based on consumption frequency rather than quantitative measures such as portion size or total fiber intake. While frequency data reflect habitual eating patterns in real-life settings, they may not fully capture variations in the amount consumed, preparation methods, or accompanying foods, which could influence glycemic outcomes.

Participants demonstrated moderate knowledge across two domains, dietary fiber sources and dietary fiber health benefits, comparable to the Saudi study on obesity/CVD/diabetes prevention knowledge (33).

The most frequent obstacle was gastrointestinal side effects (23.4%), more often reported by women (27.6% vs. 18.5%; *P* = 0.004). Other barriers included limited availability (15.5%; *P* = 0.400), dislike of taste (19.5%; *P* = 0.648), high cost (10.2%; *P* = 0.412), and lack of awareness of dietary fiber importance (23.5%; *P* = 0.583). Although most showed no gender differences, these barriers illustrate cultural and personal challenges that align with regional evidence on cost and access (33, 34). Women’s higher reporting of gastrointestinal symptoms may relate to hormonal influences on GI motility and visceral sensitivity (35)[Bibr CIT0036].

Crucially, both knowledge domains, dietary fiber sources and dietary fiber health benefits, were significantly associated with HbA1c; lower knowledge was associated with higher HbA1c levels, particularly within the 7–8% and > 8% categories, underscoring that knowledge is necessary but some other factors can affect dietary fiber intake. Effective glycemic control likely also depends on age, education, overall diet quality, physical activity, glucose monitoring, and adherence to medications (36, 37). The lack of significant associations between dietary fiber intake and HbA1c may reflect the complex clinical profile of the study population. A large proportion of participants had long-standing diabetes, suboptimal glycemic control, and were receiving combination therapy, which may attenuate the detectable impact of individual dietary components in cross-sectional analyses. Although these factors were considered conceptually, no statistically significant associations were observed in multivariable analysis within this study. This highlights the complexity of glycemic regulation in real-life settings and underscores the multifactorial nature of diabetes management.

## Strengths and limitations

A key strength of this study is its distinction of knowledge into two domains and its exploration of perceived barriers within a culturally specific context, providing insights relevant to both practice and policy. Nevertheless, the findings should be interpreted with caution, as the cross-sectional design does not allow causal inference, and dietary assessment was qualitative rather than quantitative. The use of face-to-face interviews may have introduced recall and social desirability biases. Furthermore, the lack of statistically significant associations after adjustment may reflect limitations inherent to the cross-sectional design and the use of frequency-based dietary assessment rather than portion-specific measures, while some potential confounders, including physical activity, total energy intake, and cooking methods, were not fully addressed. Moreover, cultural preparation practices were not systematically captured.

## Conclusion

This study provides new evidence on dietary fiber intake, knowledge, and glycemic control among Jordanian patients with Type 2 diabetes mellitus. Although dietary fiber is recognized for improving glycemic outcomes, no significant associations were found between HbA1c and fruit, bread, or vegetable consumption, likely due to cultural dietary practices, portion sizes, preparation methods, and food quality. Participants had moderate dietary fiber knowledge, and lower knowledge was linked to higher HbA1c, highlighting that awareness alone is insufficient for glycemic control. Barriers such as gastrointestinal discomfort, limited availability, cost, and cultural preferences further affect intake. These findings underscore the need for culturally tailored education and interventions, with healthcare providers promoting practical strategies to support sustainable dietary fiber intake in Type 2 diabetes mellitus management and translating knowledge into practice.

## Data Availability

The data supporting the findings of this study are available from the corresponding author upon reasonable request.
